# Fragmentation mass spectra dataset of linear cyanopeptides - microginins

**DOI:** 10.1016/j.dib.2020.105825

**Published:** 2020-06-10

**Authors:** Sevasti – Kiriaki Zervou, Triantafyllos Kaloudis, Anastasia Hiskia, Hanna Mazur-Marzec

**Affiliations:** a Laboratory of Photo-Catalytic Processes and Environmental Chemistry, Institute of Nanoscience & Nanotechnology, National Center for Scientific Research “Demokritos”, Patriarchou Grigoriou E & 27 Neapoleos Str, 15310 Agia Paraskevi, Athens, Greece; b Division of Marine Biotechnology, University of Gdansk, Al. Marszałka Piłsudskiego 46, 81-378 Gdynia, Poland

**Keywords:** Microginins, Fragmentation mass spectra, Structural elucidation, LC-qTRAP MS/MS, Bioactive linear cyanopeptides

## Abstract

Microginins are the less common class of bioactive linear cyanobacterial peptides. Recently, an investigation for their presence in cyanobacteria from Greek freshwaters and strain cultures was carried out. The present dataset is related to the research article “New microginins from cyanobacteria of Greek freshwaters” [1]. Cyanobacterial biomass from bloom samples and cultured strains were extracted with aqueous methanol. Extracts were analysed by liquid chromatography coupled to hybrid triple quadrupole/linear ion trap mass spectrometer (LC-qTRAP MS/MS) in information dependent acquisition (IDA) mode. Enhanced ion product (EIP) mode was applied for the collection of ion fragmentation spectra. Identification of microginins was based on the characteristic fragment ions of the unique microginin amino acid 3-amino-2-hydroxy-decanoic acid (Ahda) and its modified forms. The analysis of fragmentation spectra revealed 51 microginin structures, including 36 new variants. This article provides the dataset of fragmentation mass spectra of the microginins detected in cyanobacteria from Greek freshwaters. As this class of cyanopeptides is produced by cyanobacteria from different geographical regions, the aim of this dataset is to enable the identification of microginins in future studies and therefore to contribute to a better evaluation of their presence in freshwater bodies worldwide.

Specifications table

**Table:** 

Subject	Environmental Chemistry
Specific subject area	Bioactive cyanobacterial metabolites – class of linear peptides, microginins
Type of data	Figures
How data were acquired	Data was acquired using an Agilent 1200, high-performance liquid chromatography (HPLC) apparatus (Agilent Technologies, Waldboronn, Germany) coupled online to a hybrid triple quadrupole/linear ion trap mass spectrometer (QTRAP5500, Applied Biosystems, Sciex; Concorde, Ontario, Canada). Data acquisition and processing were accomplished using Analyst QS® 1.5.1 software.
Data format	Raw and Filtered Data
Parameters for data collection	Microginins were extracted from lyophilized cyanobacterial cells
Description of data collection	Information dependent acquisition (IDA) mode was applied for the detection of microginins and ion fragmentation spectra were collected in enhanced ion product (EIP) mode.
Data source location	Division of Marine Biotechnology, University of Gdansk, Gdansk, Poland
Data accessibility	With the article
Related research article	Sevasti – Kiriaki Zervou, Spyros Gkelis, Triantafyllos Kaloudis, Anastasia Hiskia, Hanna Mazur-Marzec, New microginins from cyanobacteria of Greek freshwaters, Chemosphere, Volume 248, June 2020, 125961 https://doi.org/10.1016/j.chemosphere.2020.125961

## Value of the Data

• The data represents a useful library of fragmentation mass spectra for a large number of microginins.
• Due to the absence of analytical standards for microginins, the dataset can be used as reference for their identification through LC-MS/MS analysis by research groups working in this field.
• The mass spectral dataset will enable studies related to the occurrence of microginins and investigations into their bio-activities.
• The dataset can also be used in retrospective mass spectral analysis of cyanobacterial biomass or bloom samples to reveal the presence of microginins.
• The provided dataset sheds light to the metabolomic potential of cyanobacteria.
• The dataset presented in this article is a useful tool for research groups in the field of environmental chemistry, biology and biochemistry, especially for those working on cyanobacterial bioactive metabolites. Identification of microginins will also enable their isolation and purification to be used as reference standards for water laboratories and lake authorities.

## 1 Data Description

The dataset contains the fragmentation mass spectra obtained during the study of occurrence of microginins in cyanobacterial bloom samples and strain cultures from Greek freshwaters [1]. Samples were analysed using a hybrid triple quadrupole/linear ion trap mass spectrometer after liquid chromatography separation (LC- qTRAP MS/MS) and the fragmentation spectra of a large number of microginins - including new variants - were obtained.

In total, fifty-one microginins’ structures were elucidated and their mass fragmentation spectra as well as their structural formula are included in the dataset (Fig. 1, Fig. 2, Fig. 3, Fig. 4, Fig. 5, Fig. 6, Fig. 7, Fig. 8, Fig. 9, Fig. 10, Fig. 11, Fig. 12, Fig. 13, Fig. 14, Fig. 15, Fig. 16, Fig. 17, Fig. 18, Fig. 19, Fig. 20, Fig. 21, Fig. 22, Fig. 23, Fig. 24, Fig. 25, Fig. 26, Fig. 27, Fig. 28, Fig. 29, Fig. 30, Fig. 31, Fig. 32, Fig. 33, Fig. 34, Fig. 35, Fig. 36, Fig. 37, Fig. 38, Fig. 39, Fig. 40, Fig. 41, Fig. 42, Fig. 43, Fig. 44, Fig. 45, Fig. 46, Fig. 47). The spectra of two microginins with new proposed 3-amino-2-hydroxy-decanoic acid (Ahda) modification, and two microginins with not fully elucidated structures, which are included in the research article [1] are not presented here. The presented microginin variants consist of four or five amino acids. Identification and structural elucidation of the peptides were based on characteristic fragment ions of Ahda moiety, immonium ions, characteristic losses of C-terminal amino acid, and other diagnostic ions related to amino acids sequence. Diagnostic fragment ions formed by Ahda and its variants were *m/z* 128 for Ahda fragment (C_8_H_18_N), *m/z* 142 for MeAhda fragment (C_9_H_20_N) [2], *m/z* 162 for Cl-Ahda fragment (C_8_H_17_NCl) and *m/z* 196 for Cl_2_-Ahda fragment (C_8_H_16_NCl_2_) [3]. Distinction between methyltyrosine (MeTyr) and homotyrosine (Htyr), which are isobaric compounds having the same chemical formula (C_10_H_13_NO_3_) could not be carried out using the applied MS/MS methodology. The same stands for amino acids leucine (Leu) and isoleucine (Ile) and their methylated units. For this reason, both amino acids are annotated in the given sequences. Further details on the structure elucidation process are described in [1].

**Fig. 1:**
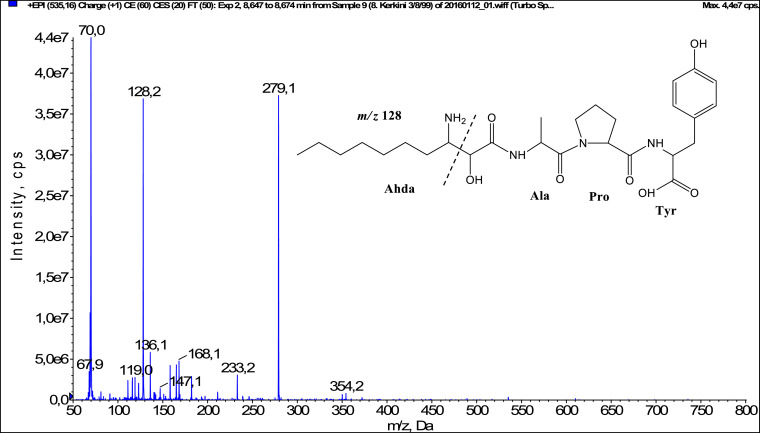
Fragmentation mass spectrum of Microginin 535 with pseudomolecular ion at *m/z* 535 [M+H]^+^ and proposed structure of the peptide: Ahda-Ala-Pro-Tyr (*m/z* 70 = Pro immonium ion, *m/z* 136 = Tyr immonium ion, *m/z* 128 = Ahda characteristic fragment ion, *m/z* 168 = [Ahda-H_2_O]^+^, *m/z* 233 = [Pro+Tyr+H-CO-H_2_O]^+^, *m/z* 279 = [Pro+Tyr+H]^+^, *m/z* 354 = [M+H-Tyr]^+^).

**Fig. 2:**
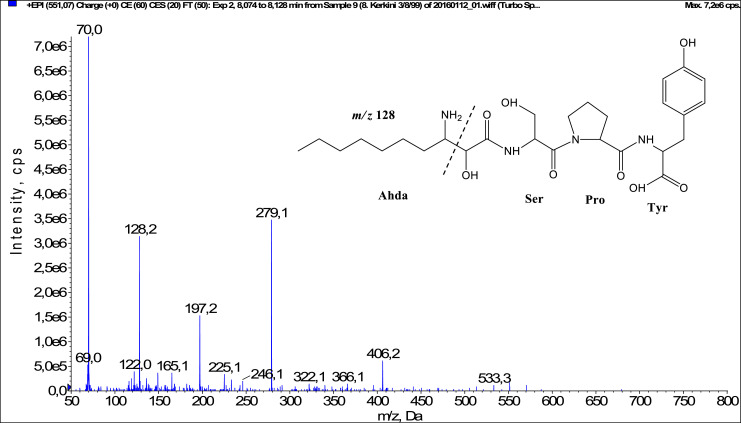
Fragmentation mass spectrum of Microginin 551 with pseudomolecular ion at *m/z* 551 [M+H]^+^ and proposed structure of the peptide: Ahda-Ser-Pro-Tyr (*m/z* 70 = Pro immonium ion, *m/z* 128 = Ahda characteristic fragment ion, *m/z* 225 = [C_2_H_2_O_2_(part of Ahda)+Ser+Pro+H-H_2_O]^+^, *m/z* 279 = [Pro+Tyr+H]^+^, *m/z* 406 = [C_2_H_2_O_2_(part of Ahda)+Ser+Pro+Tyr+H-H_2_O]^+^).

**Fig. 3:**
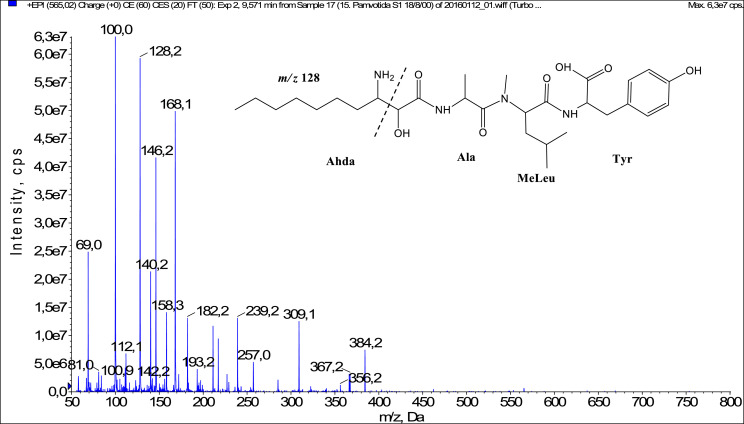
Fragmentation mass spectrum of Microginin 565A with pseudomolecular ion at *m/z* 565 [M+H]^+^ and proposed structure of the peptide: Ahda-Ala-MeLeu-Tyr (*m/z* 100 = MeLeu immonium ion, *m/z* 112 = [C_2_H_2_O_2_(part of Ahda)+Ala+H-H_2_O]^+^, *m/z* 128 = Ahda characteristic fragment ion, *m/z* 158 = [Ahda-CO]^+^, *m/z* 168 = [Ahda-H_2_O]^+^, *m/*z 182 = [Tyr+H]^+^, *m/z* 239 = [Ahda+Ala-H_2_O]^+^ or [C_2_H_2_O_2_(part of Ahda)+Ala+MeLeu+H-H_2_O]^+^, *m/z* 309 = [MeLeu+Tyr+H]^+^, *m/z* 384 = [M+H-Tyr]^+^).

**Fig. 4:**
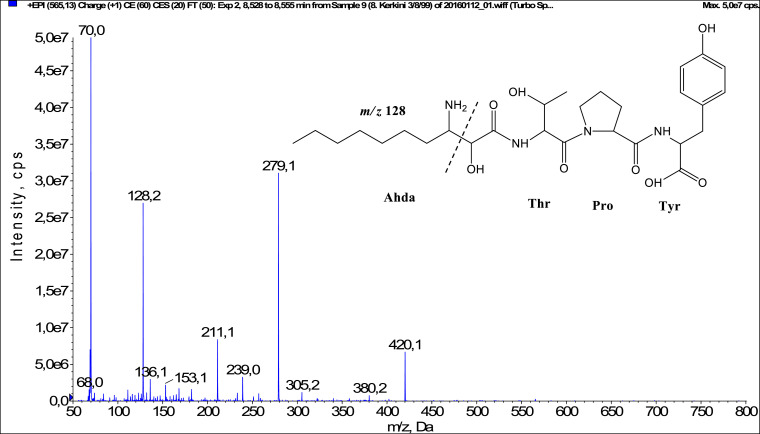
Fragmentation mass spectrum of Microginin 565B with pseudomolecular ion at *m/z* 565 [M+H]^+^ and proposed structure of the peptide: Ahda-Thr-Pro-Tyr (*m/z* 70 = Pro immonium ion, *m/z* 128 = Ahda characteristic fragment ion, *m/z* 136 = Tyr immonium ion, *m/z* 211 = [C_2_H_2_O_2_(part of Ahda)+Thr+Pro+H-H_2_O-CO]^+^, *m/z* 239 = [C_2_H_2_O_2_(part of Ahda)+Thr+Pro+H-H_2_O]^+^, *m/z* 279 = [Pro+Tyr+H]^+^, *m/z* 420 = [C_2_H_2_O_2_(part of Ahda)+Thr+Pro+Tyr+H-H_2_O]^+^).

**Fig. 5:**
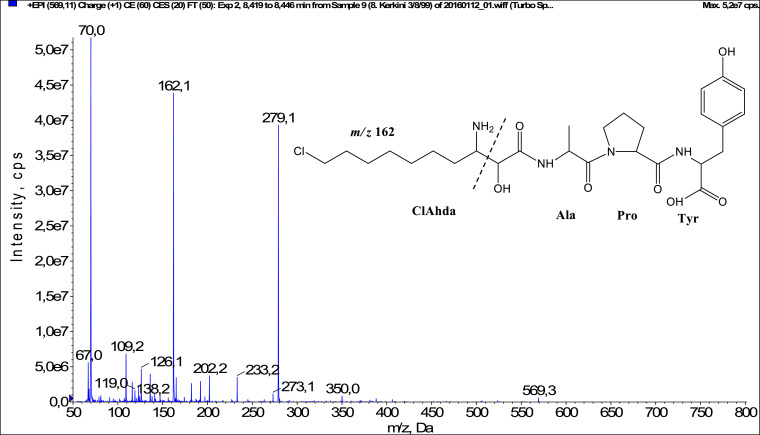
Fragmentation mass spectrum of Microginin 568 with pseudomolecular ion at *m/z* 569 [M+H]^+^ and proposed structure of the peptide: ClAhda-Ala-Pro-Tyr (*m/z* 70 = Pro immonium ion, *m/z* 162 = ClAhda characteristic fragment ion, *m/z* 279 = [Pro+Tyr+H]^+^, *m/z* 350 = [Ala+Pro+Tyr+H]^+^).

**Fig. 6:**
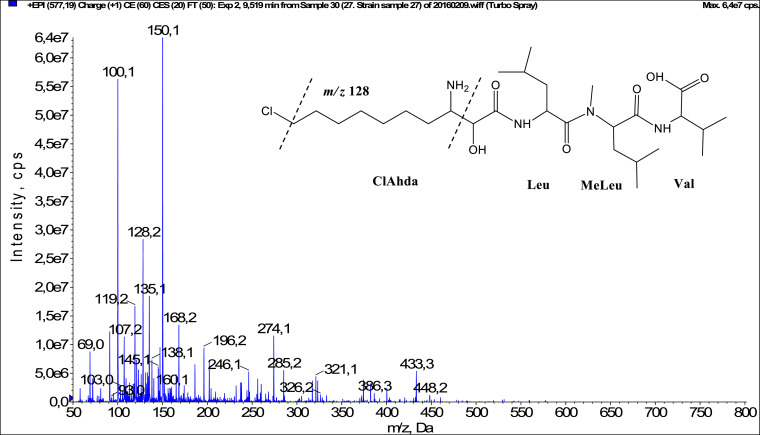
Fragmentation mass spectrum of Microginin 576 with pseudomolecular ion at *m/z* 577 [M+H]^+^ and proposed structure of the peptide: ClAhda-Leu/Ile-MeLeu/MeIle-Val (*m/z* 69 = Val immonium ion, *m/z* 100 = MeLeu immonium ion, *m/z* 128 = ClAhda fragment ion, *m/z* 433 = [M+H-Val-CO]^+^).

**Fig. 7:**
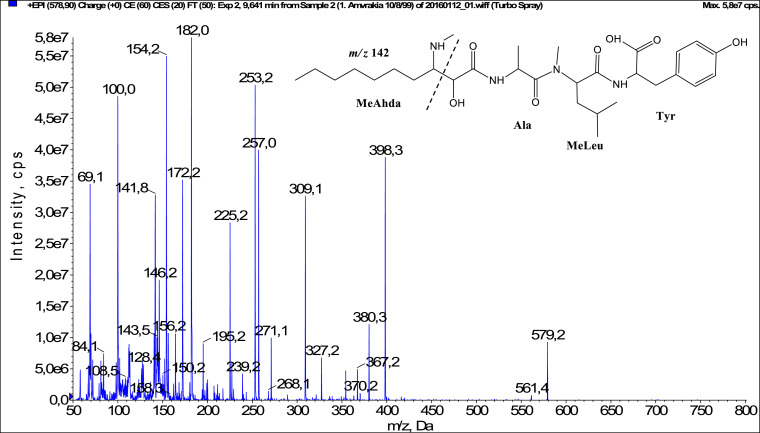
Fragmentation mass spectrum of Microginin 579A with pseudomolecular ion at *m/z* 579 [M+H]^+^ and proposed structure of the peptide: MeAhda-Ala-MeLeu/MeIle-Tyr (*m/z* 100 = MeLeu immonium ion, *m/z* 142 = MeAhda characteristic fragment ion, *m/z* 172 = [MeAhda-CO]^+^, *m/z* 182 = [MeAhda-H_2_O]^+^, *m/z* 239 = [C_2_H_2_O_2_(part of MeAhda)+Ala+MeLeu+H-H_2_O]^+^, *m/z* 253 = [M+H-MeLeu-Tyr-H_2_O]^+^, *m/z* 257 = [C_2_H_2_O_2_(part of MeAhda)+Ala+MeLeu+H]^+^, *m/z* 309 = [MeLeu+Tyr+H]^+^, *m/z* 398 = [M+H-Tyr]^+^).

**Fig. 8:**
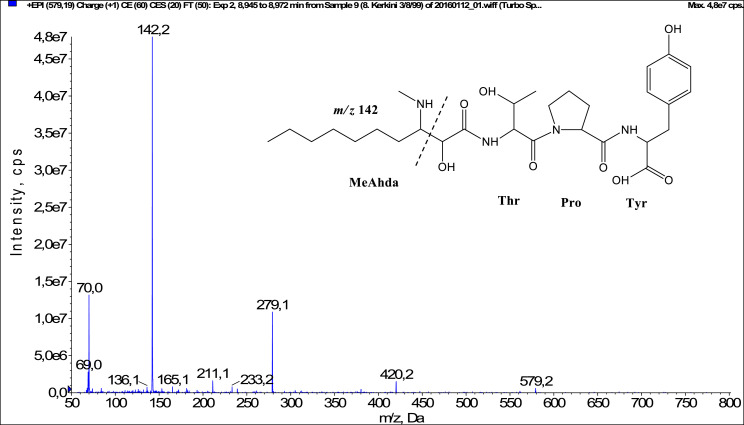
Fragmentation mass spectrum of Microginin 579B with pseudomolecular ion at *m/z* 579 [M+H]^+^ and proposed structure of the peptide: MeAhda-Thr-Pro-Tyr (*m/z* 70 = Pro immonium ion, *m/z* 136 = Tyr immonium ion, *m/z* 142 = MeAhda characteristic fragment ion, *m/z* 233 = [Pro+Tyr+H-CO-H_2_O]^+^, *m/z* 279 = [Pro+Tyr+H]^+^, *m/z* 420 = [C_2_H_2_O_2_(part of MeAhda)+Thr+Pro+Tyr+H-H_2_O]^+^).

**Fig. 9:**
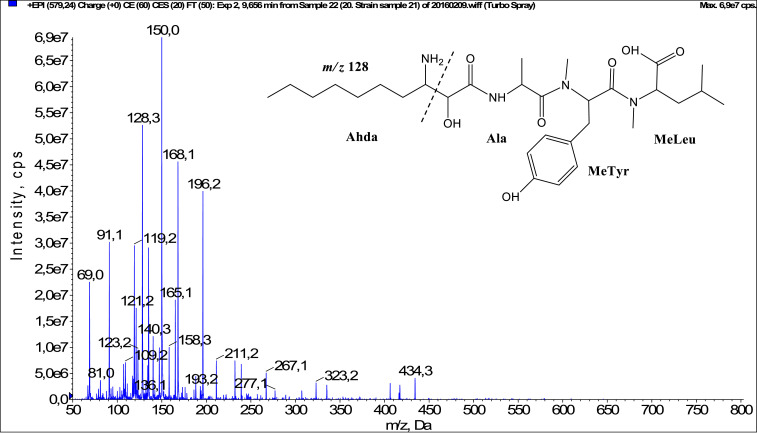
Fragmentation mass spectrum of Microginin 579C with pseudomolecular ion at *m/z* 579 [M+H]^+^ and proposed structure of the peptide: Ahda-Ala-MeTyr/Htyr-MeLeu/MeIle (*m/z* 128 = Ahda characteristic fragment ion, *m/z* 150 = MeTyr immonium ion, *m/z* 158 = [Ahda-CO]^+^, *m/z* 168 = [Ahda-H_2_O]^+^, *m/z* 323 = [MeTyr+MeLeu+H]^+^, *m/z* 434 = [M+H-MeLeu]^+^).

**Fig. 10:**
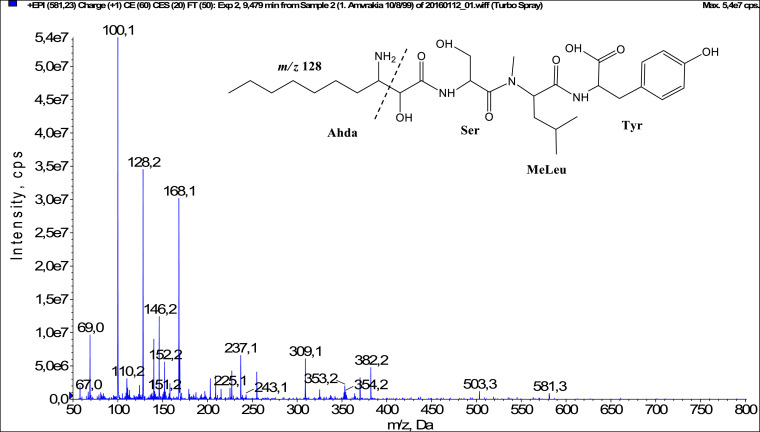
Fragmentation mass spectrum of Microginin 581 with pseudomolecular ion at *m/z* 581 [M+H]^+^ and proposed structure of the peptide: Ahda-Ser-MeLeu/MeIle-Tyr (*m/z* 100 = MeLeu immonium ion, *m/z* 128 = Ahda characteristic fragment ion, *m/z* 168 = [Ahda-H_2_O]^+^, *m/z* 309 = [MeLeu+Tyr+H]^+^, *m/z* 354 = [M+H-Tyr-H_2_O-CO]^+^, *m/z* 382 = [M+H-Tyr-H_2_O]^+^).

**Fig. 11:**
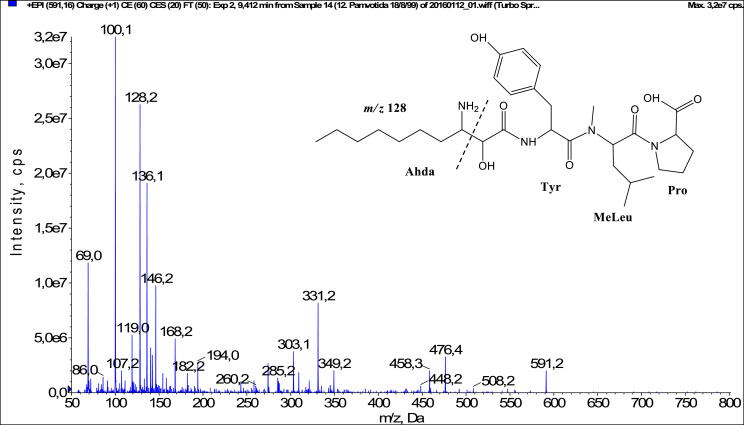
Fragmentation mass spectrum of Microginin 591B with pseudomolecular ion at *m/z* 591 [M+H]^+^ and proposed structure of the peptide: Ahda-Tyr-MeLeu/MeIle-Pro (*m/z* 100 = MeLeu immonium ion, *m/z* 128 = Ahda characteristic fragment ion, *m/z* 136 = Tyr immonium ion, *m/z* 168 = [Adha-H_2_O]^+^, *m/z* 194 = [C_2_H_2_O_2_(part of Ahda)+Tyr+H-CO]^+^, *m/z* 303 = [M+H-MeLeu-Pro-CO-H_2_O]^+^, *m/z* 331 = [M+H-MeLeu-Pro-H_2_O]^+^, *m/z* 349 = [C_2_H_2_O_2_(part of Ahda)+Tyr+MeLeu+H]^+^, *m/z* 448 = [M+H-Pro-CO]^+^, *m/z* 458 = [M+H-Pro-H_2_O]^+^, *m/z* 476 = [M+H-Pro]^+^).

**Fig. 12:**
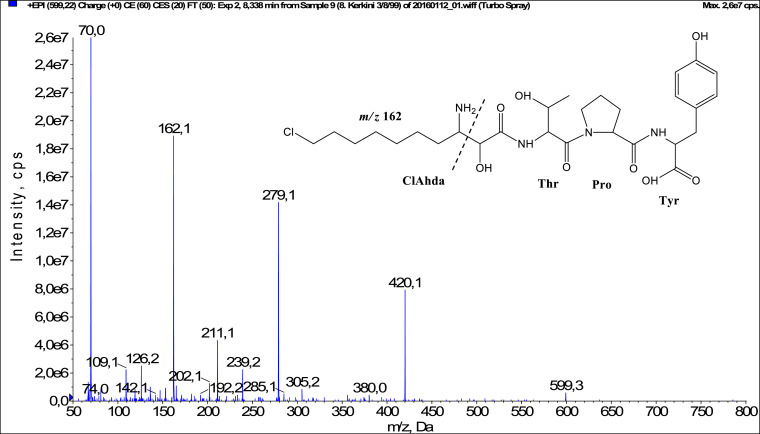
Fragmentation mass spectrum of Microginin 598 with pseudomolecular ion at *m/z* 599 [M+H]^+^ and proposed structure of the peptide: ClAhda-Thr-Pro-Tyr (*m/z* 70 = Pro immonium ion, *m/z* 162 = ClAhda characteristic fragment ion, *m/z* 192 = [ClAhda-CO]^+^, *m/z* 202 [ClAhda-H_2_O]^+^, *m/z* 239 = [C_2_H_2_O_2_(part of ClAhda)+Thr+Pro+H-H_2_O]^+^, *m/z* 279 = [Pro+Tyr+H]^+^, *m/z* 420 = [C_2_H_2_O_2_(part of ClAhda)+Thr+Pro+Tyr+H-H_2_O]^+^).

**Fig. 13:**
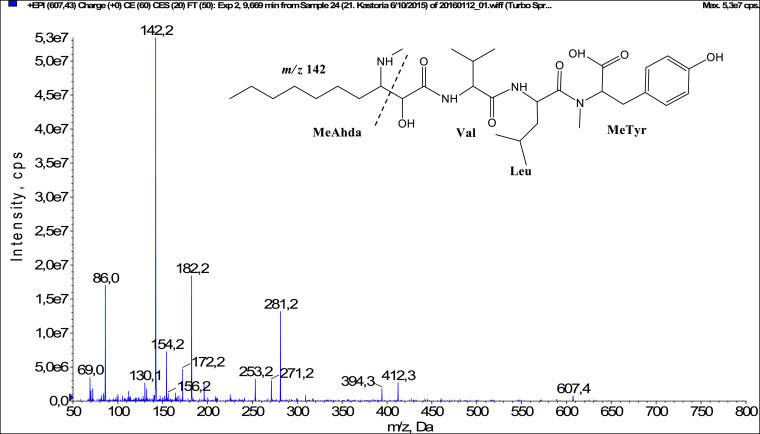
Fragmentation mass spectrum of Microginin 607A with pseudomolecular ion at *m/z* 607 [M+H]^+^ and proposed structure of the peptide: MeAhda-Val-Leu/Ile-Metyr/Htyr (*m/z* 86 = Leu immonium ion, *m/z* 142 = MeAhda characteristic fragment ion, *m/z* 172 = [MeAhda-CO]^+^, *m/z* 182 = [MeAhda-H_2_O]^+^, *m/z* 271 = [MeAhda+Val-CO]^+^, *m/z* 281 = [MeAhda+Val-H_2_O]^+^, *m/z* 394 = [M+H-MeTyr-CO]^+^, *m/z* 412 = [M+H-MeTyr]^+^).

**Fig. 14:**
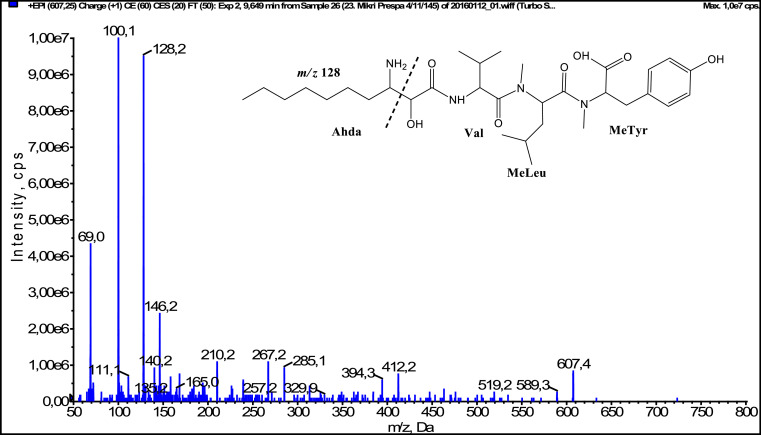
Fragmentation mass spectrum of Microginin 607B with pseudomolecular ion at *m/z* 607 [M+H]^+^ and proposed structure of the peptide: Ahda-Val-MeLeu/MeIle-MeTyr/Htyr (*m/z* 100 = MeLeu immonium ion, *m/z* 128 = Ahda characteristic fragment ion, *m/z* 267 = [Ahda+Val+H-H_2_O]^+^, *m/z* 285 = [Ahda+Val+H]^+^, *m/z* 394 = [M+H-MeTyr-H_2_O]^+^, *m/z* 412 = [M+H-MeTyr]^+^).

**Fig. 15:**
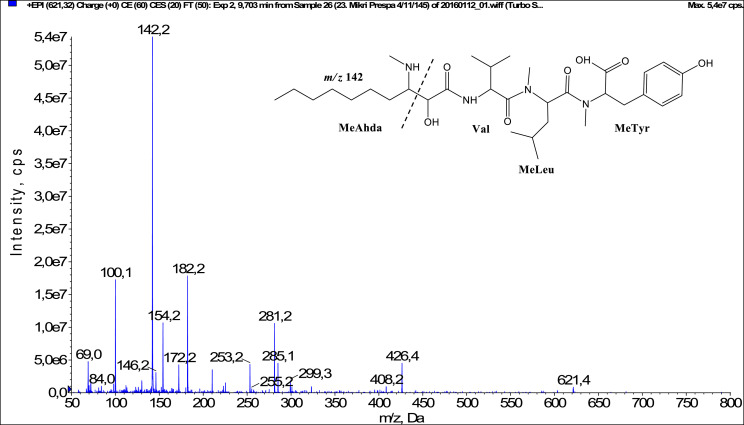
Fragmentation mass spectrum of Microginin 621A with pseudomolecular ion at *m/z* 621 [M+H]^+^ and proposed structure of the peptide: MeAhda-Val-MeLeu/MeIle-MeTyr/Htyr (*m/z* 100 = MeLeu immonium ion, *m/z* 142 = MeAhda characteristic fragment ion, *m/z* 172 = [MeAhda-CO]^+^, *m/z* 182 = [MeAhda-H_2_O]^+^, *m/z* 253 = [MeAhda+Val-CO-H_2_O]^+^, *m/z* 281 = [MeAhda+Val-H_2_O]^+^, *m/z* 426 = [M+H-MeTyr]^+^).

**Fig. 16:**
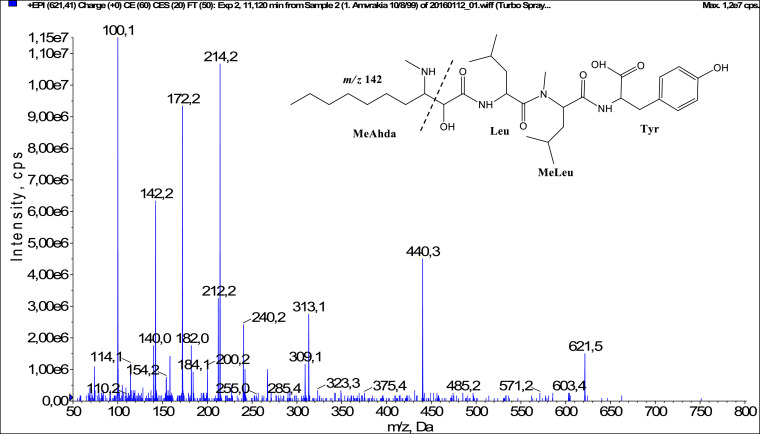
Fragmentation mass spectrum of Microginin 621B with pseudomolecular ion at *m/z* 621 [M+H]^+^ and proposed structure of the peptide: MeAhda-Leu/Ile-MeLeu/MeIle-Tyr (*m/z* 100 = MeLeu immonium ion, *m/z* 142 = MeAhda characteristic fragment ion, *m/z* 172 = [MeAhda-CO]^+^, *m/z* 182 = [MeAhda-H_2_O]^+^, *m/z* 212 = [Leu+MeLeu-CO]^+^, *m/z* 240 = [Leu+MeLeu]^+^, *m/z* 309 = [MeLeu+Tyr+H]^+^, *m/z* 313 = [M+H-MeLeu-Tyr]^+^, *m/z* 440 = [M+H-Tyr]^+^).

**Fig. 17:**
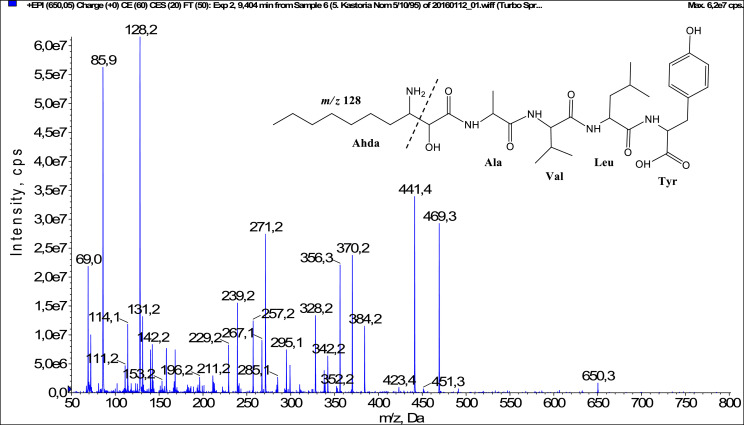
Fragmentation mass spectrum of Microginin 650 with pseudomolecular ion at *m/z* 650 [M+H]^+^ and proposed structure of the peptide: Ahda-Ala-Val-Leu/Ile-Tyr (*m/z* 86 = Leu immonium ion, *m/z* 128 = Ahda characteristic fragment ion, *m/z* 239 = [Ahda+Ala-H_2_O]^+^, *m/z* 257 = [M+H-Val-Leu-Tyr]^+^, *m/z* 295 = [Leu+Tyr+H]^+^, *m/z* 328 = [M+H-Leu-Tyr-CO]^+^, *m/z* 342 = [C_2_H_2_O_2_(part of Ahda)+Ala+Val+Leu+H]^+^, *m/z* 356 = [M+H-Leu-Tyr]^+^, *m/z* 441 = [M+H-Tyr-CO]^+^, *m/z* 469 = [M+H-Tyr]^+^).

**Fig. 18:**
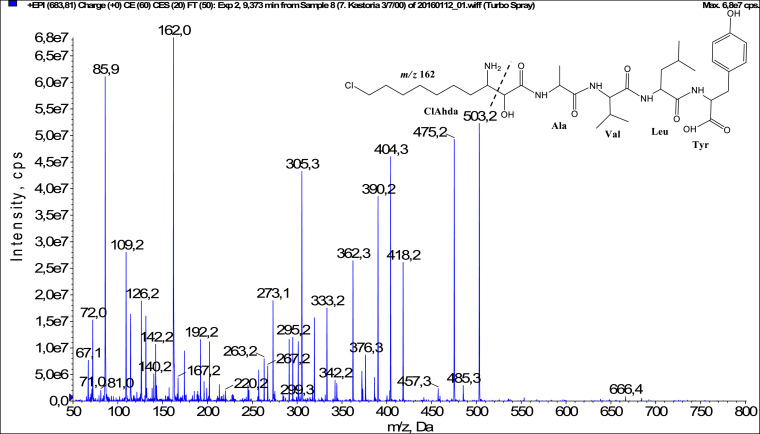
Fragmentation mass spectrum of Microginin 683 with pseudomolecular ion at *m/z* 684 [M+H]^+^ and proposed structure of the peptide: ClAhda-Ala-Val-Leu/Ile-Tyr (*m/z* 86 = Leu immonium ion, *m/z* 162 = ClAhda characteristic fragment ion, *m/z* 263 = [ClAhda+Ala-CO]^+^, *m/z* 273 = [ClAhda+Ala-H_2_O]^+^, *m/z* 229 = [C_2_H_2_O_2_(part of ClAhda)+Ala+Val+H]^+^, *m/z* 295 = [Leu+Tyr+H]^+^, *m/z* 342 = [C_2_H_2_O_2_(part of ClAhda)+Ala+Val+Leu+H]^+^, *m/z* 362 = [M+H-Leu-Tyr-CO]^+^, *m/z* 390 = [M+H-Leu-Tyr]^+^, *m/z* 503 = [M+H-Tyr]^+^).

**Fig. 19:**
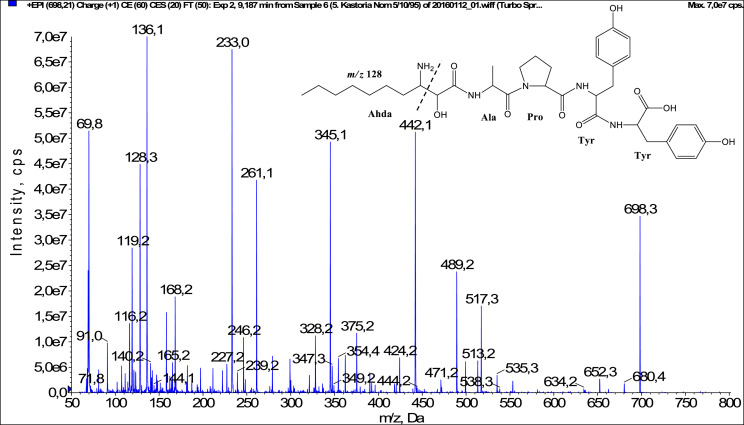
Fragmentation mass spectrum of Microginin T2 with pseudomolecular ion at *m/z* 698 [M+H]^+^ and proposed structure of the peptide: Ahda-Ala-Pro-Tyr-Tyr (*m/z* 70 = Pro immonium ion, *m/z* 128 = Ahda characteristic fragment ion, *m/z* 136 = Tyr immonium ion, *m/z* 168 = [Ahda-H_2_O]^+^, *m/z* 227 = [C_2_H_2_O_2_(part of Ahda)+Ala+Pro+H]^+^, *m/z* 233 = [Pro+Tyr+H-CO]^+^, *m/z* 261 = [Pro+Tyr+H]^+^, *m/z* 345 = [Tyr+Tyr+H]^+^, *m/z* 442 = [Pro+Tyr+Tyr+H]^+^, *m/z* 489 = [M+H-Tyr-CO]^+^, *m/z* 517 = [M+H-Tyr]^+^).

**Fig. 20:**
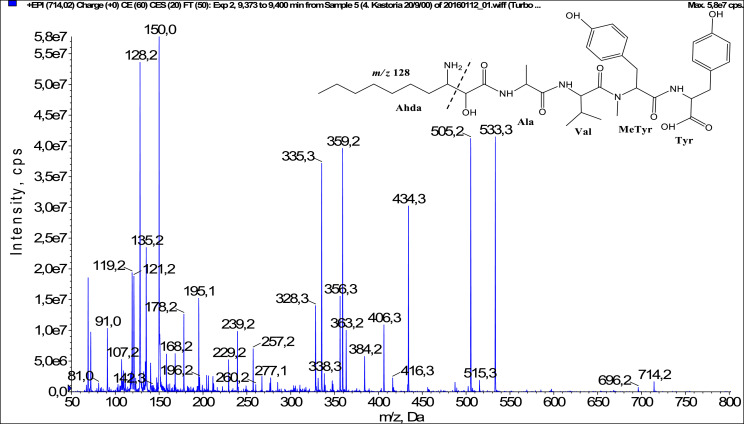
Fragmentation mass spectrum of Microginin with pseudomolecular ion at *m/z* 714 [M+H]^+^ and proposed structure of the peptide: Ahda-Ala-Val-MeTyr-Tyr (*m/z* 128 = Ahda characteristic fragment ion, *m/z* 150 = MeTyr immonium ion, *m/z* 229 = [C_2_H_2_O_2_(part of Ahda)+Ala+Val+H]^+^, *m/z* 257 = [Ahda+Ala+H]^+^, *m/z* 328 = [Ahda+Ala+Val+H-CO]^+^, *m/z* 338 = [Ahda+Ala+Val+H-H_2_O]^+^, *m/z* 356 = [Ahda+Ala+Val+H]^+^, *m/z* 359 = [MeTyr+Tyr+H]^+^, *m/z* 406 = [C_2_H_2_O_2_(part of Ahda)+Ala+Val+MeTyr+H]^+^, *m/z* 505 = [M+H-Tyr-CO]^+^, *m/z* 533 = [M+H-Tyr]^+^).

**Fig. 21:**
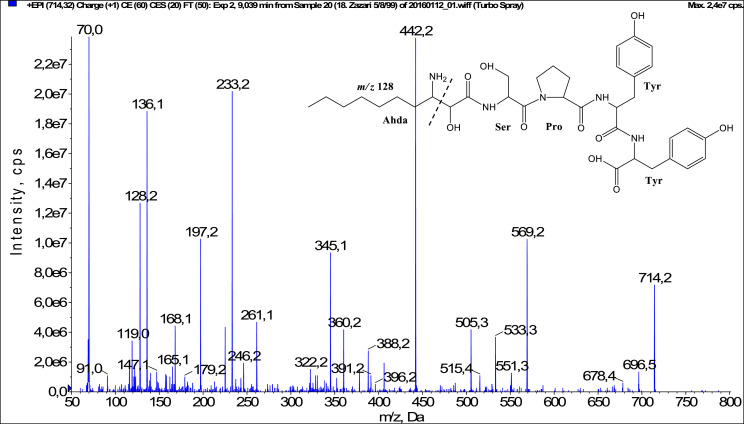
Fragmentation mass spectrum of Microginin 714B with pseudomolecular ion at *m/z* 714 [M+H]^+^ and proposed structure of the peptide: Ahda-Ser-Pro-Tyr-Tyr (*m/z* 70 = Pro immonium ion, *m/z* 128 = Ahda characteristic fragment ion, *m/z* 136 = Tyr immonium ion, *m/z* 168 = [Ahda-H_2_O]^+^, *m/z* 233 = [Pro+Tyr+H-CO]^+^, *m/z* 261 = [Pro+Tyr+H]^+^, *m/z* 345 = [Tyr+Tyr+H]^+^, *m/z* 442 = [Pro+Tyr+Tyr+H]^+^, *m/z* 505 = [M+H-Tyr-CO]^+^, *m/z* 533 = [M+H-Tyr]^+^).

**Fig. 22:**
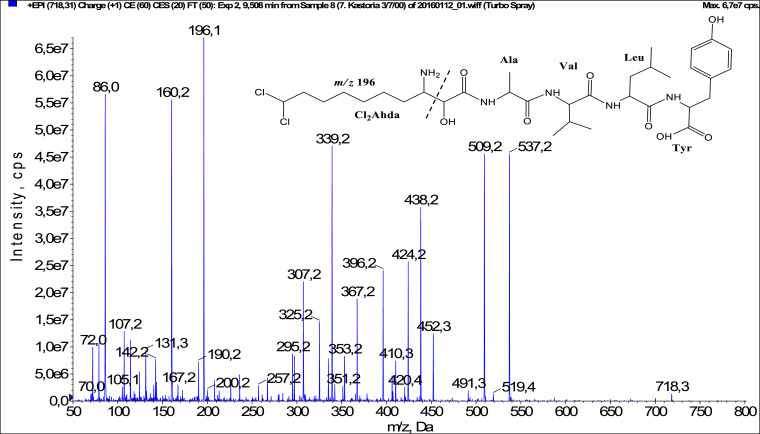
Fragmentation mass spectrum of Microginin 717 with pseudomolecular ion at *m/z* 718 [M+H]^+^ and proposed structure of the peptide: Cl_2_Ahda-Ala-Val-Leu/Ile-Tyr (*m/z* 86 = Leu immonium ion, *m/z* 160 = [Cl_2_Ahda fragment-HCl]^+^, *m/z* 196 = Cl_2_Ahda characteristic fragment ion, *m/z* 307 = [Cl_2_Ahda+Ala-H_2_O]^+^, *m/z* 325 = [Cl_2_Ahda+Ala]^+^, *m/z* 424 = [M+H-Leu-Tyr]^+^, *m/z* 509 = [M+H-Tyr-CO]^+^, *m/z* 537 = [M+H-Tyr]^+^).

**Fig. 23:**
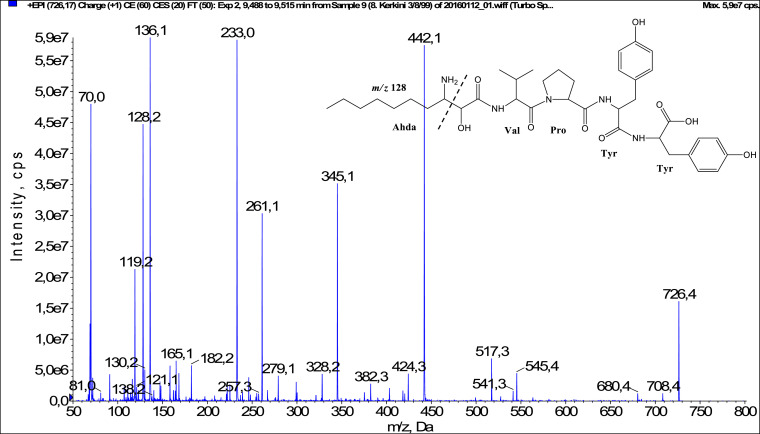
Fragmentation mass spectrum of Microginin FR5 with pseudomolecular ion at *m/z* 726 [M+H]^+^ and proposed structure of the peptide: Ahda-Val-Pro-Tyr-Tyr (*m/z* 70 = Pro immonium ion, *m/z* 128 = Ahda characteristic fragment ion, *m/z* 136 = Tyr immonium ion, *m/z* 233 = [Pro+Tyr+H-CO]^+^, *m/z* 261 = [Pro+Tyr+H]^+^, *m/z* 345 = [Tyr+Tyr+H]^+^, *m/z* 424 = [Pro+Tyr+Tyr+H-H_2_O]^+,^*m/z* 442 = [Pro+Tyr+Tyr+H]^+^, *m/z* 517 = [M+H-Tyr-CO]^+^, *m/z* 545 = [M+H-Tyr]^+^).

**Fig. 24:**
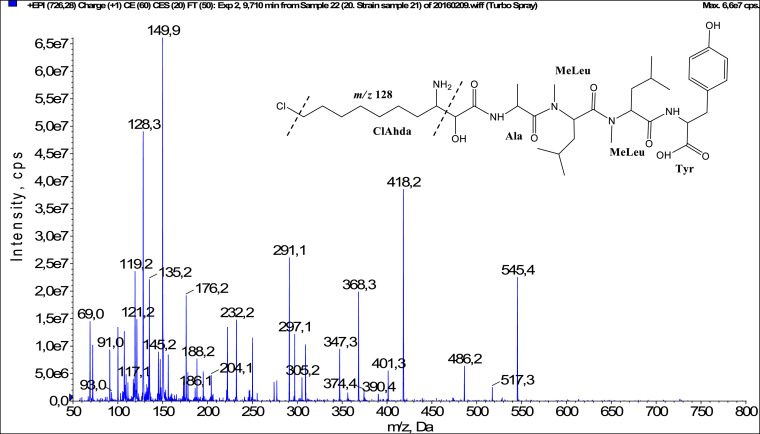
Fragmentation mass spectrum of Microginin 725 with pseudomolecular ion at *m/z* 726 [M+H]^+^ and proposed structure of the peptide: ClAhda-Ala-MeLeu/MeIle-MeLeu/MeIle-Tyr (*m/z* 100 = MeLeu immonium ion, *m/z* 128 = ClAhda fragment ion, *m/z* 291 = [M+H-MeLeu-MeLeu-Tyr]^+^, *m/z* 418 = [M+H-MeLeu-Tyr]^+^, *m/z* 517 = [M+H-Tyr-CO]^+^, *m/z* 545 = [M+H-Tyr]^+^).

**Fig. 25:**
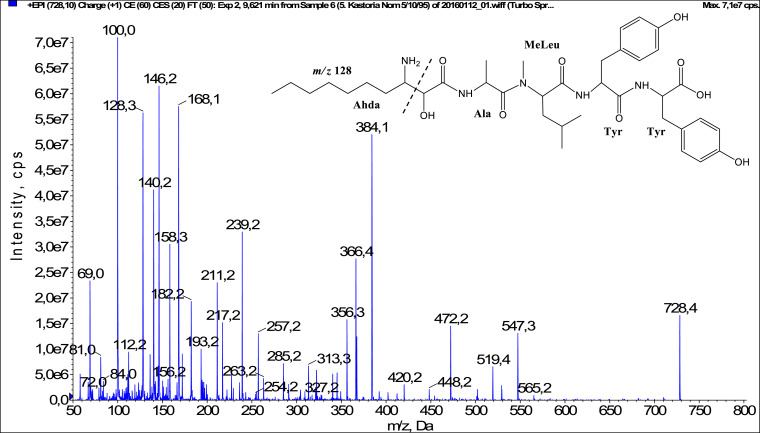
Fragmentation mass spectrum of Microginin FR1 with pseudomolecular ion at *m/z* 728 [M+H]^+^ and proposed structure of the peptide: Ahda-Ala-MeLeu-Tyr-Tyr (*m/z* 100 = MeLeu immonium ion, *m/z* 128 = Ahda characteristic fragment ion, *m/z* 158 = [Ahda-CO]^+^, *m/z* 168 = [Ahda-H_2_O]^+^, *m/z* 239 = [C_2_H_2_O_2_(part of Ahda)+Ala+MeLeu+H-H_2_O]^+^, *m/z* 257 = [C_2_H_2_O_2_(part of Ahda)+Ala+MeLeu+H]^+^, *m/z* 356 = [M+H-Tyr-Tyr-CO]^+^, *m/z* 366 = [M+H-Tyr-Tyr-H_2_O]^+^, *m/z* 384 = [M+H-Tyr-Tyr]^+^, *m/z* 547 = [M+H-Tyr]^+^).

**Fig. 26:**
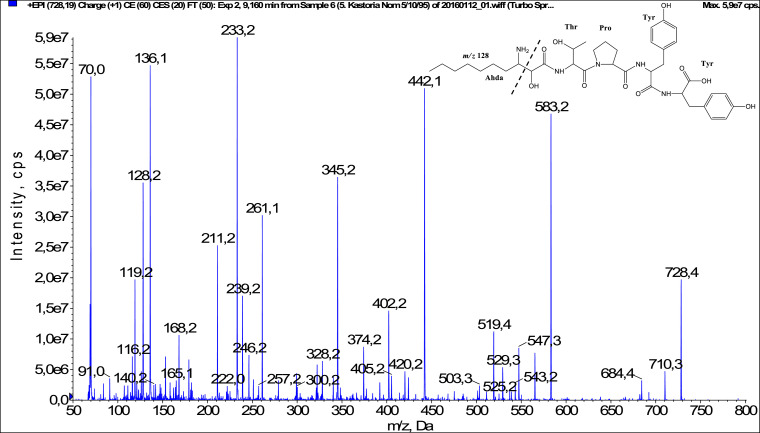
Fragmentation mass spectrum of Microginin FR3 with pseudomolecular ion at *m/z* 728 [M+H]^+^ and proposed structure of the peptide: Ahda-Thr-Pro-Tyr-Tyr (*m/z* 70 = Pro immonium ion, *m/z* 128 = Ahda characteristic fragment ion, *m/z* 136 = Tyr immonium ion, *m/z* 233 = [Pro+Tyr+H-CO]^+^, *m/z* 261 = [Pro+Tyr+H]^+^, *m/z* 345 = [Tyr+Tyr+H]^+^, *m/z* 442 = [Pro+Tyr+Tyr+H]^+^, *m/z* 547 = [M+H-Tyr]^+^, *m/z* 583 = [M+H-Ahda fragment-H_2_O]^+^).

**Fig. 27:**
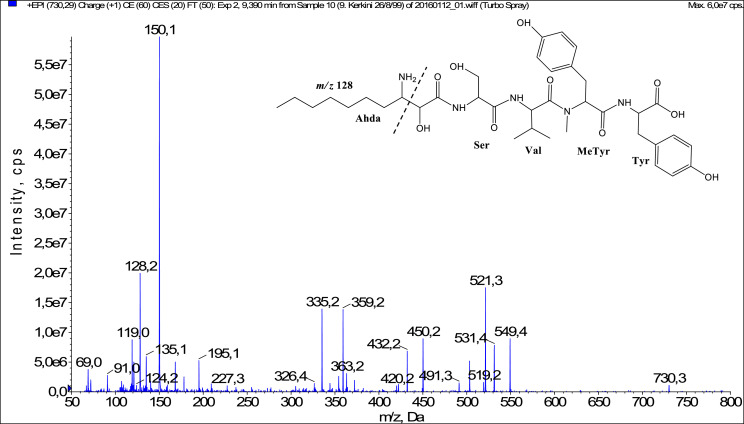
Fragmentation mass spectrum of Microginin 730 with pseudomolecular ion at *m/z* 730 [M+H]^+^ and proposed structure of the peptide: Ahda-Ser-Val-MeTyr/Htyr-Tyr (*m/z* 128 = Ahda characteristic fragment ion, *m/z* 150 = MeTyr immonium ion, *m/z* 335 = [Ser+Val+MeTyr-CO]^+^, *m/z* 359 = [MeTyr+Tyr+H]^+^, *m/z* 364 = [Ser+Val+MeTyr+H]^+^, *m/z* 521 = [M+H-Tyr-CO], *m/z* 549 = [M+H-Tyr]^+^).

**Fig. 28:**
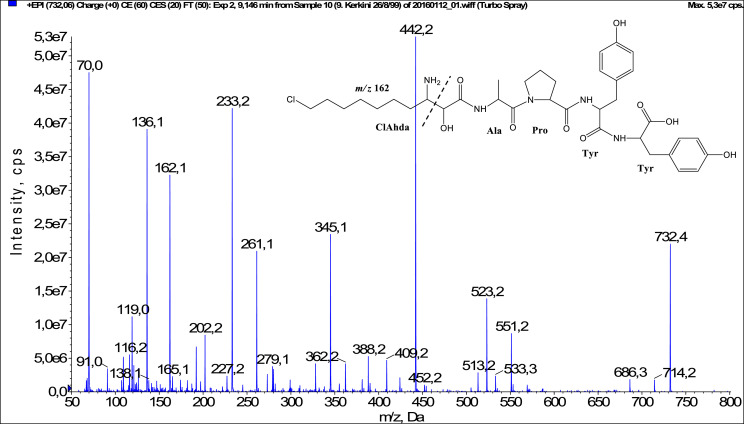
Fragmentation mass spectrum of Microginin T1 with pseudomolecular ion at *m/z* 732 [M+H]^+^ and proposed structure of the peptide: ClAhda-Ala-Pro-Tyr-Tyr (*m/z* 70 = Pro immonium ion, *m/z* 136 = Tyr immonium ion, *m/z* 162 = ClAhda characteristic fragment ion, *m/z* 233 = [Pro+Tyr+H-CO]^+^, *m/z* 261 = [Pro+Tyr+H]^+^, *m/z* 345 = [Tyr+Tyr+H]^+^, *m/z* 388 = [M+H-Tyr-Tyr]^+^, *m/z* 442 = [Pro+Tyr+Tyr+H]^+^, *m/z* 523 = [M+H-Tyr-CO]^+^, *m/z* 533 = [M+H-Tyr-H_2_O]^+^, *m/z* 551 = [M+H-Tyr]^+^).

**Fig. 29:**
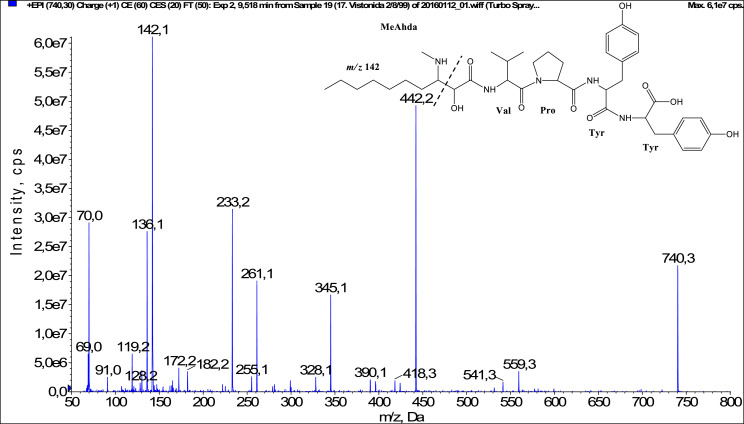
Fragmentation mass spectrum of Microginin FR6 with pseudomolecular ion at *m/z* 740 [M+H]^+^ and proposed structure of the peptide: MeAhda-Val-Pro-Tyr-Tyr (*m/z* 70 = Pro immonium ion, *m/z* 136 = Tyr immonium ion, *m/z* 142 = MeAhda characteristic fragment ion, *m/z* 172 = [MeAhda-CO]^+^, *m/z* 182 = [MeAhda-H_2_O]^+^, *m/z* 233 = [Pro+Tyr+H-CO]^+^, *m/z* 261 = [Pro+Tyr+H]^+^, *m/z* 345 = [Tyr+Tyr+H]^+^, *m/z* 442 = [Pro+Tyr+Tyr+H]^+^, *m/z* 541 = [M+H-Tyr-H_2_O]^+^, *m/z* 559 = [M+H-Tyr]^+^).

**Fig. 30:**
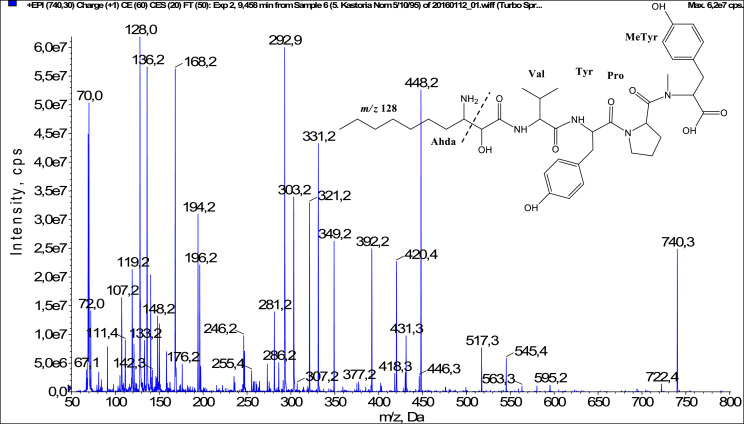
Fragmentation mass spectrum of Microginin 740B with pseudomolecular ion at *m/z* 740 [M+H]^+^ and proposed structure of the peptide: Ahda-Val-Tyr-Pro-MeTyr/Htyr (*m/z* 70 = Pro immonium ion, *m/z* 128 = Ahda characteristic fragment ion, *m/z* 136 = Tyr immonium ion, *m/z* 168 = [Ahda-H_2_O]^+^, *m/z* 293 = [C_2_H_2_O_2_(part of Ahda)+Val+Tyr+H-CO]^+^, *m/z* 303 = [C_2_H_2_O_2_(part of Ahda)+Val+Tyr+H-H_2_O]^+^, *m/z* 321 = [C_2_H_2_O_2_(part of Ahda)+Val+Tyr+H]^+^, *m*/z 420 = [M+H-Pro-MeTyr-CO]^+^, *m/z* 448 = [M+H-Pro-MeTyr]^+^, *m/z* 517 = [M+H-MeTyr-CO]^+^, *m/z* 545 = [M+H-MeTyr]^+^).

**Fig. 31:**
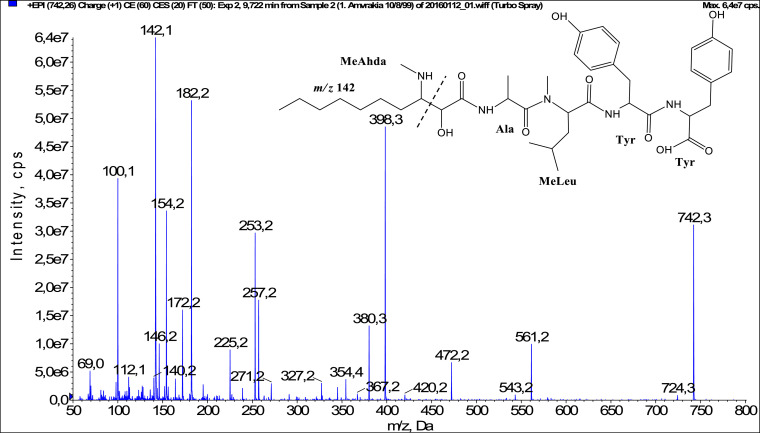
Fragmentation mass spectrum of Microginin 742A with pseudomolecular ion at *m/z* 742 [M+H]^+^ and proposed structure of the peptide: MeAhda-Ala-MeLeu/MeIle-Tyr-Tyr (*m/z* 100 = MeLeu immonium ion, *m/z* 142 = MeAhda characteristic fragment ion, *m/z* 172 = [MeAhda-CO]^+^, *m/z* 182 = [MeAhda-H_2_O]^+^, *m/z* 253 = [M+H-MeLeu-Tyr-Tyr-H_2_O]^+^, *m/z* 257 = [C_2_H_2_O_2_(part of MeAhda)+Ala+MeLeu+H]^+^, *m/z* 380 = [M+H-Tyr-Tyr-H_2_O]^+^, *m/z* 398 = [M+H-Tyr-Tyr]^+^, *m/z* 420 = [C_2_H_2_O_2_(part of MeAhda)+Ala+MeLeu+Tyr+H]^+^, *m/z* 472 = [MeLeu+Tyr+Tyr+H]^+^, *m/z* 561 = [M+H-Tyr]^+^).

**Fig. 32:**
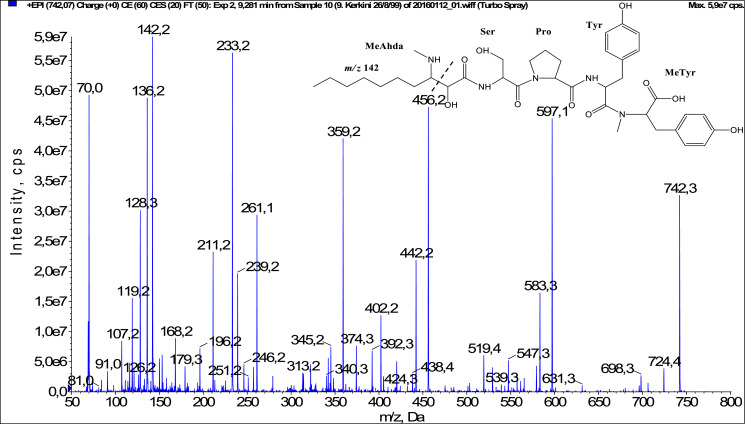
Fragmentation mass spectrum of Microginin 742B with pseudomolecular ion at *m/z* 742 [M+H]^+^ and proposed structure of the peptide: MeAhda-Ser-Pro-Tyr-MeTyr/Htyr (*m/z* 70 = Pro immonium ion, *m/z* 136 = Tyr immonium ion, *m/z* 142 = MeAhda characteristic fragment ion, *m/z* 233 = [Pro+Tyr+H-CO]^+^, *m/z* 261 = [Pro+Tyr+H]^+^, *m/z* 359 = [Tyr+MeTyr+H]^+^, *m/z* 456 = [Pro+Tyr+MeTyr+H]^+^, *m/z* 547 = [M+H-MeTyr]^+^).

**Fig. 33:**
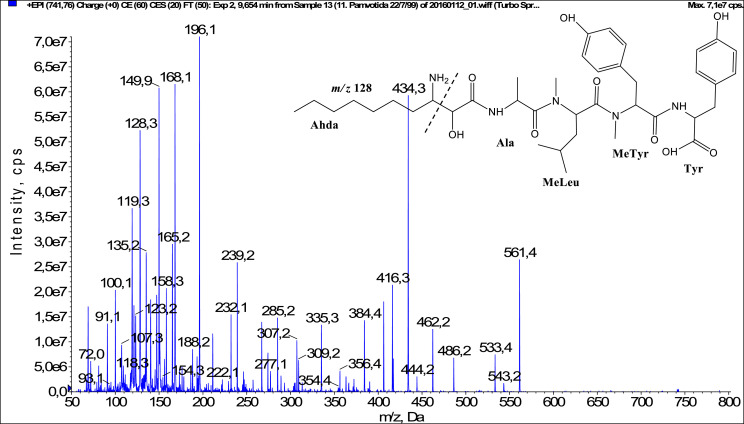
Fragmentation mass spectrum of Microginin742C with pseudomolecular ion at *m/z* 742 [M+H]^+^ and proposed structure of the peptide: Ahda-Ala-MeLeu/MeIle-MeTyr/Htyr-Tyr (*m/z* 100 = MeLeu immonium ion, *m/z* 128 = Ahda characteristic fragment ion, *m/z* 239 = [C_2_H_2_O_2_(part of Ahda)+Ala+MeLeu+H-H_2_O]^+^, *m/z* 384 = [M+H-MeTyr-Tyr]^+^, *m/z* 416 = [C_2_H_2_O_2_(part of Ahda)+Ala+MeLeu+MeTyr+H-H_2_O]^+^, *m/z* 434 = [C_2_H_2_O_2_(part of Ahda)+Ala+MeLeu+MeTyr+H]^+^, *m/z* 533 = [M+H-Tyr-CO]^+^, *m/z* 561 = [M+H-Tyr]^+^).

**Fig. 34:**
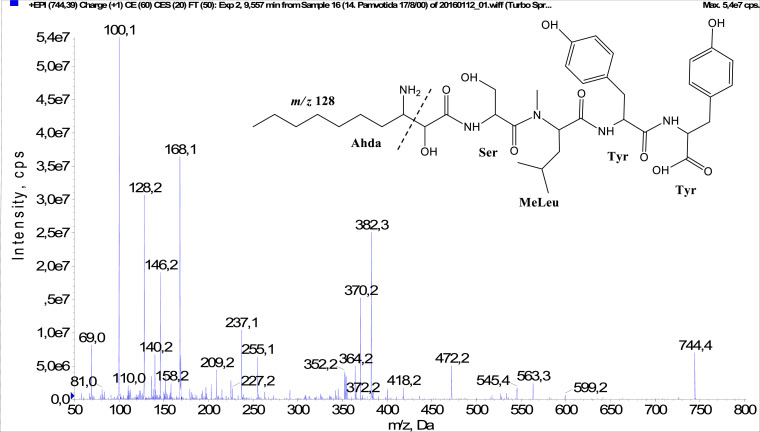
Fragmentation mass spectrum of Microginin 744 with pseudomolecular ion at *m/z* 744 [M+H]^+^ and proposed structure of the peptide: Ahda-Ser-MeLeu/MeIle-Tyr-Tyr (*m/z* 100 = MeLeu immonium ion, *m/z* 128 = Ahda characteristic fragment ion, *m/z* 146 = [C_2_H_2_O_2_(part of Ahda)+Ser+H]^+^, *m/z* 158 = [Ahda-CO]^+^, *m/z* 168 = [Ahda-H_2_O]^+^, *m/z* 382 = [Ahda+Ser+MeLeu-H_2_O]^+^, *m/z* 418 = [C_2_H_2_O_2_(part of Ahda)+Ser+MeLeu+Tyr+H-H_2_O]^+^, *m/z* 472 = [MeLeu+Tyr+Tyr+H]^+^, *m/z* 563 = [M+H-Tyr]^+^).

**Fig. 35:**
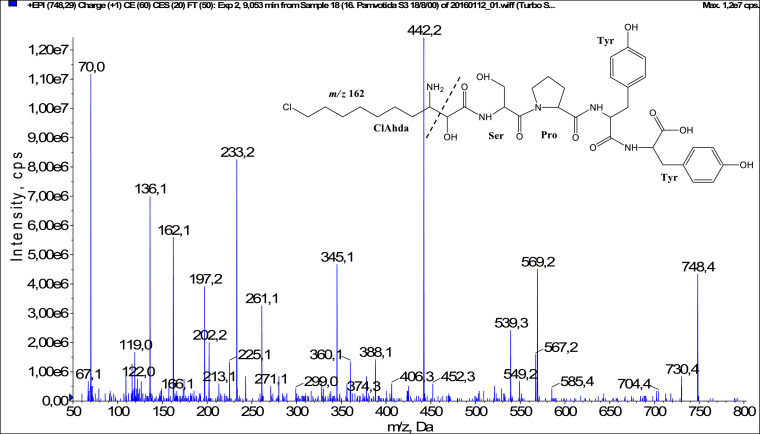
Fragmentation mass spectrum of Microginin 747A with pseudomolecular ion at *m/z* 748 [M+H]^+^ and proposed structure of the peptide: ClAhda-Ser-Pro-Tyr-Tyr (*m/z* 70 = Pro immonium ion, *m/z* 136 = Tyr immonium ion, *m/z* 162 = ClAhda characteristic fragment ion, *m/z* 233 = [Pro+Tyr+H-CO]^+^, *m/z* 261 = [Pro+Tyr+H]^+^, *m/z* 345 = [Tyr+Tyr+H]^+^, *m/z* 388 = [C_2_H_2_O_2_(part of ClAhda)+Ser+Pro+Tyr+H-H_2_O]^+^, *m/z* 406 = [C_2_H_2_O_2_(part of ClAhda)+Ser+Pro+Tyr+H]^+^, *m/z* 442 = [Pro+Tyr+Tyr+H]^+^, *m/z* 539 = [M+H-Tyr-CO]^+^, *m/z* 549 = [M+H-Tyr-H_2_O]^+^, *m/z* 567 = [M+H-Tyr]^+^).

**Fig. 36:**
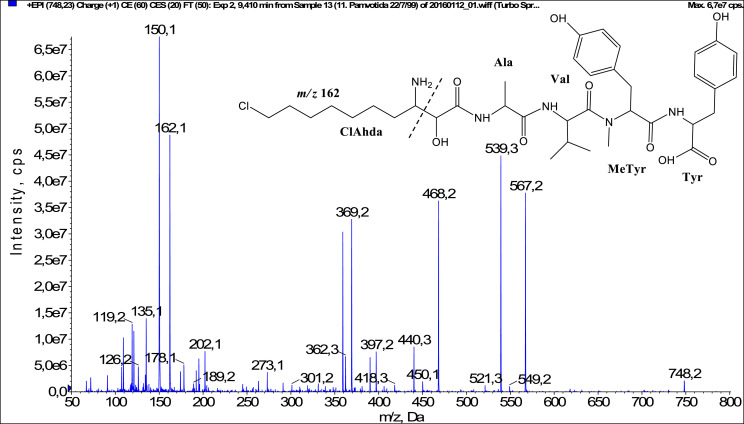
Fragmentation mass spectrum of Microginin 747B with pseudomolecular ion at *m/z* 748 [M+H]^+^ and proposed structure of the peptide: ClAhda-Ala-Val-MeTyr/Htyr-Tyr (*m/z* 150 = MeTyr immonium ion, *m/z* 162 = ClAhda characteristic fragment ion, *m/z* 359 = [Ala+Val+MeTyr+H_2_O+H]^+^, *m/z* 362 = [M+H-MeTyr-Tyr-CO]^+^, *m/z* 539 = [M+H-Tyr-CO]^+^, *m/z* 567 = [M+H-Tyr]^+^).

**Fig. 37:**
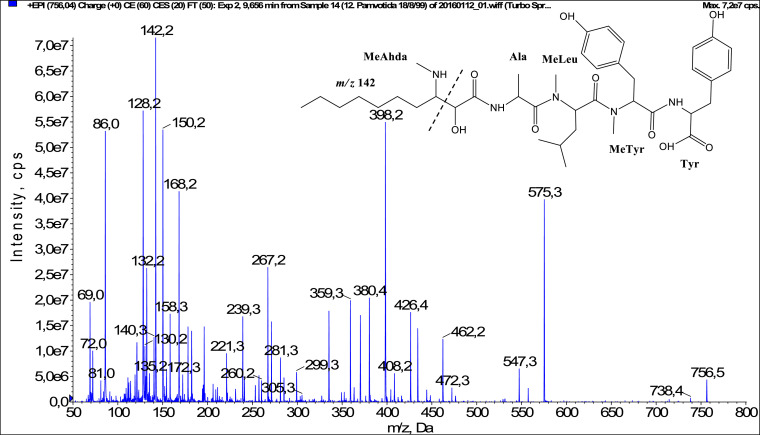
Fragmentation mass spectrum of Microginin 756B with pseudomolecular ion at *m/z* 756 [M+H]^+^ and proposed structure of the peptide: MeAhda-Ala-MeLeu/MeIle-MeTyr/Htyr-Tyr (*m/z* 142 = MeAhda characteristic fragment ion, *m/z* 150 = MeTyr immonium ion, *m/z* 239 = [C_2_H_2_O_2_(part of MeAhda)+Ala+MeLeu+H-H_2_O]^+^, *m/z* 359 = [MeTyr+Tyr+H]^+^, *m/z* 380 = [M+H-MeTyr-Tyr-H_2_O]^+^, *m/z* 398 = [M+H-MeTyr-Tyr]^+^, *m/z* 547 = [M+H-Tyr-CO]^+,^*m/z* 575 = [M+H-Tyr]^+^).

**Fig. 38:**
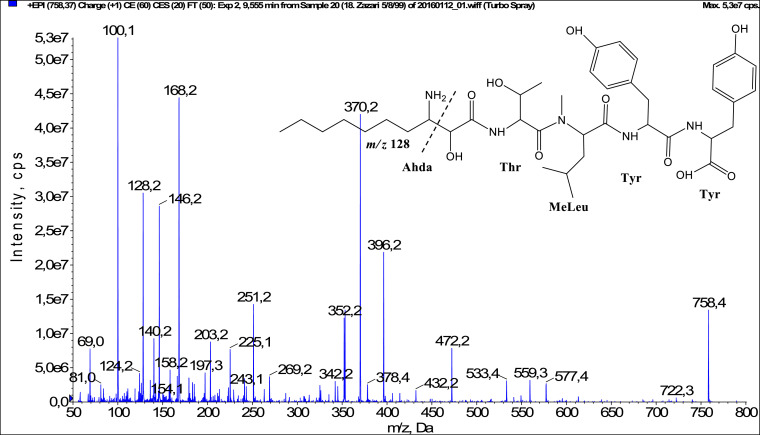
Fragmentation mass spectrum of Microginin 757 with pseudomolecular ion at *m/z* 758 [M+H]^+^ and proposed structure of the peptide: Ahda-Thr-MeLeu/MeIle-Tyr-Tyr (*m/z* 100 = MeLeu immonium ion, *m/z* 128 = Ahda characteristic fragment ion, *m/z* 158 = [Ahda-CO]^+^, *m/z* 168 = [Ahda-H_2_O]^+^, *m/z* 269 = [C_2_H_2_O_2_(part of Ahda)+Thr+MeLeu+H-H_2_O]^+^, *m/z* 396 = [Ahda+Thr+MeLeu-H_2_O]^+^, *m/z* 432 = [C_2_H_2_O_2_(part of Ahda)+Thr+MeLeu+Tyr-H_2_O]^+^, *m/z* 472 = [M+H-(Ahda+Thr)]^+^, *m/z* 559 = [M+H-Tyr-H_2_O]^+^, *m/z* 577 = [M+H-Tyr]^+^).

**Fig. 39:**
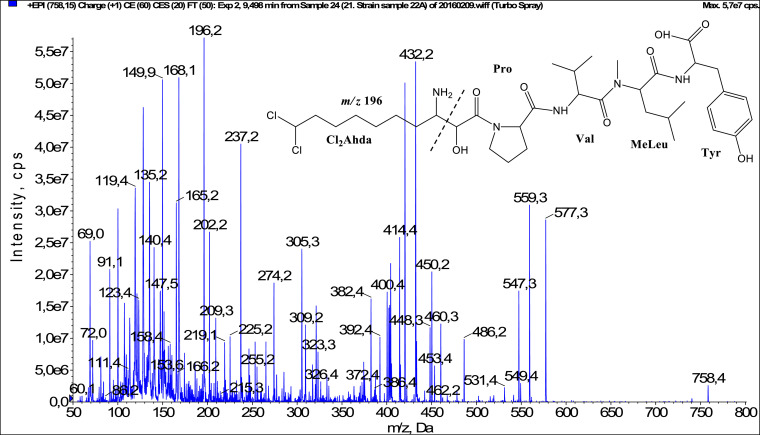
Fragmentation mass spectrum of Microginin 757B with pseudomolecular ion at *m/z* 758 [M+H]^+^ and proposed structure of the peptide: Cl_2_Ahda-Pro-Val-MeLeu/MeIle-Tyr (*m/z* 100 = MeLeu immonium ion, *m/z* 196 = Cl_2_Ahda characteristic fragment ion, *m/z* 382 = [C_2_H_2_O_2_(part of Cl_2_Ahda)+Pro+Val+MeLeu+H]^+^, *m/z* 432 = [M+H-MeLeu-Tyr-H_2_O]^+^, *m/z* 450 = [M+H-MeLeu-Tyr]^+^, *m/z* 559 = [M+H-Tyr-H_2_O]^+^, *m/z* 577 = [M+H-Tyr]^+^).

**Fig. 40:**
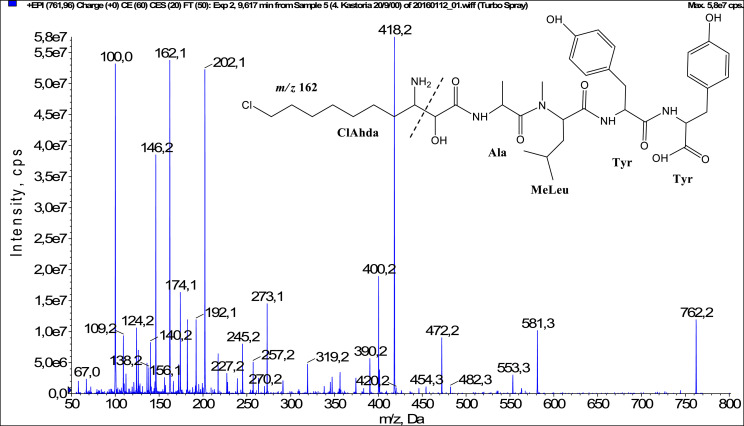
Fragmentation mass spectrum of Microginin 761A with pseudomolecular ion at *m/z* 762 [M+H]^+^ and proposed structure of the peptide: ClAhda-Ala-MeLeu/MeIle-Tyr-Tyr (*m/z* 100 = MeLeu immonium ion, *m/z* 162 = ClAhda characteristic fragment ion, *m/z* 192 = [ClAhda-CO]^+^, *m/z* 202 = [ClAhda-H_2_O]^+^, *m/z* 273 = [ClAhda+Ala-H_2_O]^+^, *m/z* 400 = [M+H-Tyr-Tyr-H_2_O]^+^, *m/z* 418 = [M+H-Tyr-Tyr]^+^, *m/z* 553 = [M+H-Tyr-CO]^+^, *m/z* 581 = [M+H-Tyr]^+^).

**Fig. 41:**
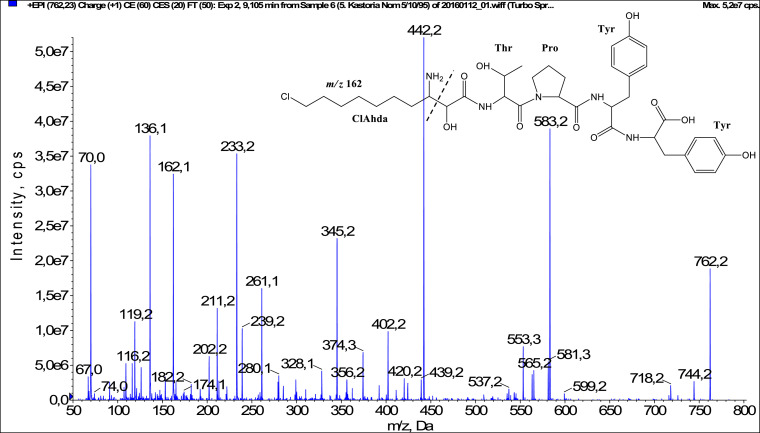
Fragmentation mass spectrum of Microginin 761B with pseudomolecular ion at *m/z* 762 [M+H]^+^ and proposed structure of the peptide: ClAhda-Thr-Pro-Tyr-Tyr (*m/z* 70 = Pro immonium ion, *m/z* 136 = Tyr immonium ion, *m/z* 162 = ClAhda characteristic fragment ion, *m/z* 233 = [Pro+Tyr+H-CO]^+^, *m/z* 261 = [Pro+Tyr+H]^+^, *m/z* 239 = [C_2_H_2_O_2_(part of ClAhda)+Thr+Pro+H-H_2_O]^+^, *m/z* 345 = [Tyr+Tyr+H]^+^, *m/z* 402 = [C_2_H_2_O_2_(part of ClAhda)+Thr+Pro+Tyr+H-H_2_O]^+^, *m/z* 420 = [C_2_H_2_O_2_(part of ClAhda)+Thr+Pro+Tyr+H]^+^, *m/z* 442 = [Pro+Tyr+Tyr+H]^+^, *m/z* 553= [M+H-Tyr-CO]^+^, *m/z* 581 = [M+H-Tyr]^+^, *m/z* 583 = [M+H-ClAhda-H_2_O]^+^).

**Fig. 42:**
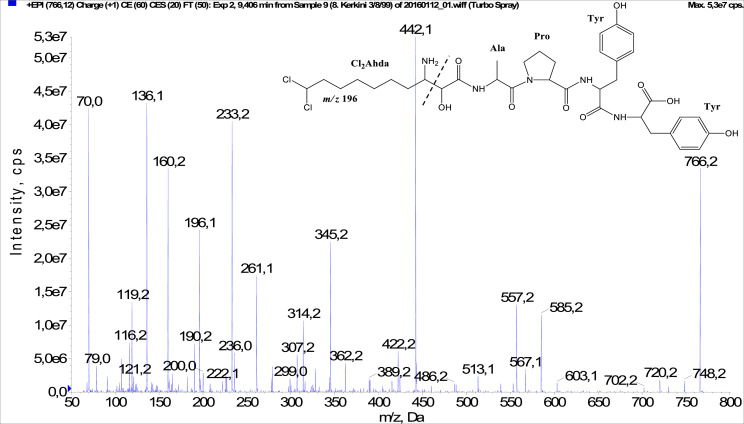
Fragmentation mass spectrum of Microginin 765 with pseudomolecular ion at *m/z* 766 [M+H]^+^ and proposed structure of the peptide: Cl_2_Ahda-Ala-Pro-Tyr-Tyr (*m/z* 70 = Pro immonium ion, *m/z* 136 = Tyr immonium ion, *m/z* 196 = Cl_2_Ahda characteristic fragment ion, *m/z* 233 = [Pro+Tyr+H-CO]^+^, *m/z* 261 = [Pro+Tyr+H]^+^, *m/z* 345 = [Tyr+Tyr+H]^+^, *m/z* 442 = [Pro+Tyr+Tyr+H]^+^, *m/z* 513 = [Ala+Pro+Tyr+Tyr+H]^+^, *m/z* 557 = [M+H-Tyr-CO]^+^, *m/z* 585 = [M+H-Tyr]^+^).

**Fig. 43:**
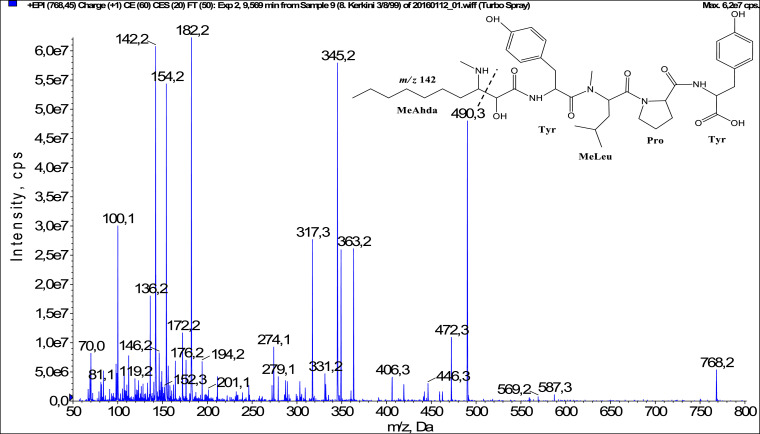
Fragmentation mass spectrum of Microginin KR767 with pseudomolecular ion at *m/z* 768 [M+H]^+^ and proposed structure of the peptide: MeAhda-Tyr-MeLeu-Pro-Tyr (*m/z* 100 = MeLeu immonium ion, *m/z* 142 = MeAhda characteristic fragment ion, *m/z* 172 = [MeAhda-CO]^+^, *m/z* 182 = [MeAhda-H_2_O]^+^, *m/z* 317 = [MeAhda+Tyr-H_2_O-CO]^+^, *m/z* 345 = [MeAhda+Tyr-H_2_O]^+^, *m/z* 472 =[M+H-Pro-Tyr-H_2_O]^+^*m/z* 490 = [M+H-Pro-Tyr]^+^, *m/z* 569 = [M+H-Tyr-H_2_O]^+^, *m/z* 587 = [M+H-Tyr]^+^).

**Fig. 44:**
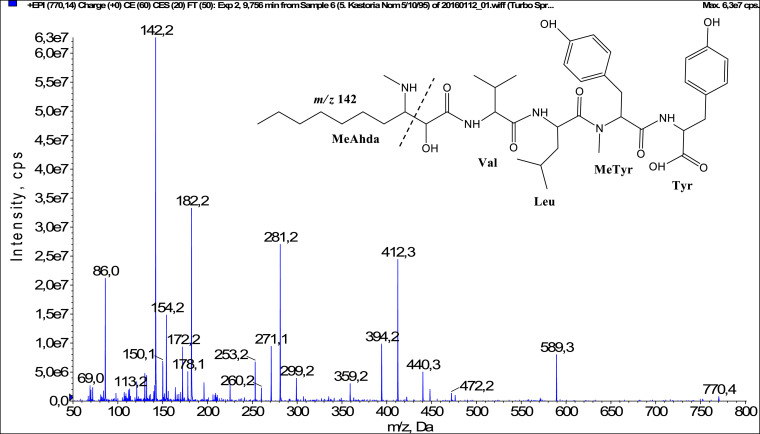
Fragmentation mass spectrum of Microginin 770 with pseudomolecular ion at *m/z* 770 [M+H]^+^ and proposed structure of the peptide: MeAhda-Val-Leu/Ile-MeTyr/Htyr-Tyr (*m/z* 86 = Leu immonium ion, *m/z* 142 = MeAhda characteristic fragment ion, *m/z* 182 = [MeAhda-H_2_O]^+^ or [Tyr+H]^+^, *m/z* 271 = [MeAhda+Val-CO]^+^, *m/z* 281 = [MeAhda+Val-H_2_O]^+^, *m/z* 299 = [MeAhda+Val]^+^, *m/z* 359 = [MeTyr+Tyr+H]^+^, *m/z* 394 = [M+H-MeTyr-Tyr-H_2_O]^+^, *m/z* 412 = [M+H-MeTyr-Tyr]^+^, *m/z* 472 = [Leu+MeTyr+Tyr+H]^+^, *m/z* 589 = [M+H-Tyr]^+^).

**Fig. 45:**
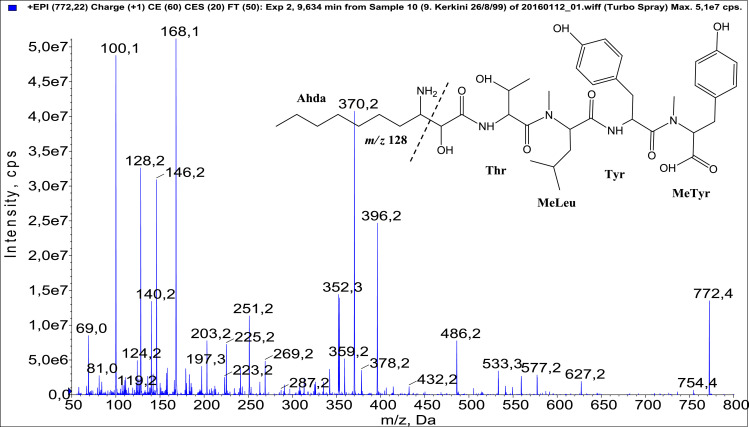
Fragmentation mass spectrum of Microginin 772 with pseudomolecular ion at *m/z* 772 [M+H]^+^ and proposed structure of the peptide: Ahda-Thr-MeLeu/MeIle-Tyr-MeTyr/Htyr (*m/z* 100 = MeLeu immonium ion, *m/z* 128 = Ahda characteristic fragment ion, *m/z* 168 = [Ahda-H_2_O]^+^, *m/z* 269 = [C_2_H_2_O_2_(part of Ahda)+Thr+MeLeu+H-H_2_O]^+^, *m/z* 287 = [C_2_H_2_O_2_(part of Ahda)+Thr+MeLeu+H]^+^, *m/z* 396 = [Ahda+Thr+MeLeu-H_2_O]^+^, *m/z* 432 = [C_2_H_2_O_2_(part of Ahda)+Thr+MeLeu+Tyr+H-H_2_O]^+^, *m/z* 486 = [MeLeu+Tyr+MeTyr+H]^+^, *m/z* 577 = [M+H-MeTyr]^+^).

**Fig. 46:**
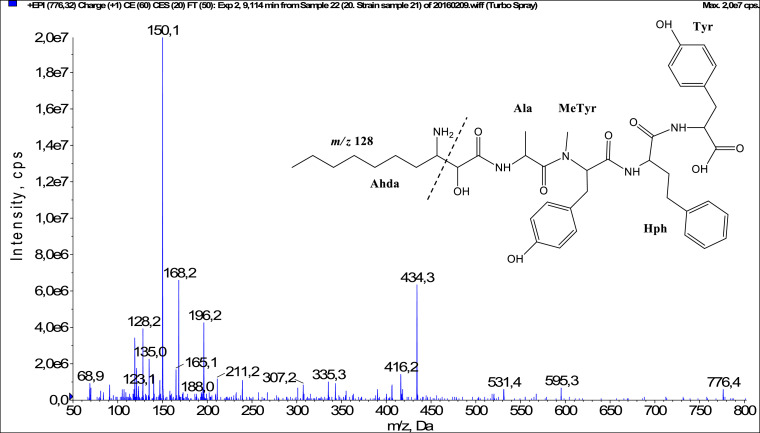
Fragmentation mass spectrum of Microginin 776 with pseudomolecular ion at *m/z* 776 [M+H]^+^ and proposed structure of the peptide: Ahda-Ala-MeTyr/Htyr-Hph-Tyr (*m/z* 128 = Ahda characteristic fragment ion, *m/z* 150 = MeTyr immonium ion, *m/z* 168 = [Ahda-H_2_O]^+^, *m/z* 307 = [C_2_H_2_O_2_(part of Ahda)+Ala+MeTyr+H]^+^, *m/z* 416 = [M+H-Hph-Tyr-H_2_O]^+^, *m/z* 434 = [M+H-Hph-Tyr]^+^, *m/z* 595 = [M+H-Tyr]^+^).

**Fig. 47:**
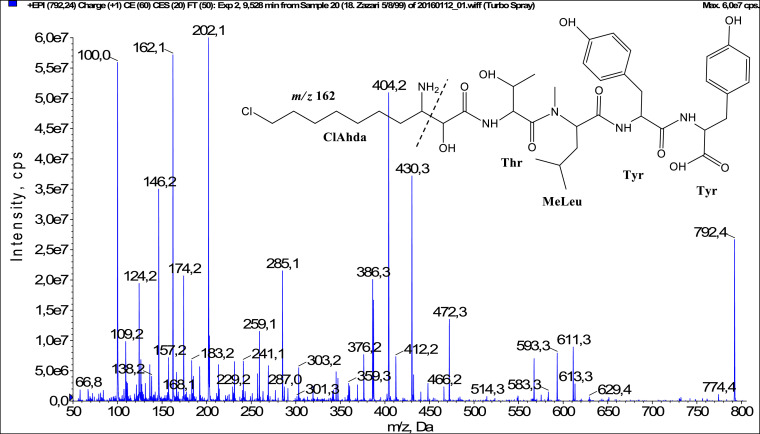
Fragmentation mass spectrum of Microginin 791 with pseudomolecular ion at *m/z* 792 [M+H]^+^ and proposed structure of the peptide: ClAhda-Thr-MeLeu/MeIle-Tyr-Tyr (*m/z* 100 = MeLeu immonium ion, *m/z* 162 = ClAhda characteristic fragment ion, *m/z* 202 = [ClAhda-H_2_O]^+^, *m/z* 269 = [C_2_H_2_O_2_(part of ClAhda)+Thr+MeLeu+H-H_2_O]^+^, *m/z* 430 = [M+H-Tyr-Tyr-H_2_O]^+^, *m/z* 472 = [M+H-(ClAhda+Thr)]^+^, *m/z* 583 = [M+H-Tyr-CO]^+^, *m/z* 593 = [M+H-Tyr-H_2_O]^+^, *m/z* 611 = [M+H-Tyr]^+^).

## 2 Experimental Design, Materials, and Methods

### 2.1 Cyanobacterial cells extraction

In order to release the cell-bond microginins, cyanobacterial cells were extracted with aqueous methanol. For this purpose, 1.5 ml of methanol:water (75%: 25%) was added to ∼10 mg of lyophilized cyanobacterial biomass and mixed thoroughly by vortexing before sonication in ice bath for 15 min. Samples were centrifuged at 10,000 rpm for 15 min. The collected supernatant extracts were additionally centrifuged at 10,000 rpm for 5 min prior to LC-MS/MS analysis.

### 2.2 LC-qTRAP MS/MS analysis

Cyanobacterial extracts were analyzed by an Agilent 1200, liquid chromatography apparatus (Agilent Technologies, Waldboronn, Germany) coupled online to a hybrid triple quadrupole/linear ion trap mass spectrometer (QTRAP5500, Applied Biosystems, Sciex; Concorde, Ontario, Canada) according to Mazur-Marzec et al. [4]. Briefly, 5 μL of extracts were injected in a reversed-phase column (Zorbax Eclipse XDB-C18, 4.6 × 150 mm, 5 μm, Agilent Technologies, Santa Clara, CA, USA) and microginins were separated within 13 min by a gradient program with mobile phase composed of (A) acetonitrile and (B) 5% acetonitrile in MilliQ water, both containing 0.1% formic acid, at a flow rate of 0.6 mL/min. Gradient program was starting with 15% A, which was linearly increased to 75% in 5 min, then to 90% in the next 5 min, held for 5 min and brought back to 15% A in 1 min for equilibration. ESI positive ionization was applied at IonSpray voltage 5500, ion source temperature 550 °C and curtain gas (CUR) 20. For the detection of microginins information dependent acquisition (IDA) mode was applied, while the ion fragmentation spectra were collected in enhanced ion product (EIP) mode. In the IDA mode, a full scan from 500 to 1200 Da was acquired and, if the signal of an ion was above a threshold of 10^4^ cps, EIP mode was triggered and the ion was fragmented in the collision cell (Q2). Fragmentation spectra were acquired from 50 to 1000 Da with a scan speed of 2000 Da/s and collision energy (CE) of 60 eV with collision energy spread (CES) of 20 eV, declustering potential (DP) 80. Data acquisition and processing were carried out using Analyst QS® 1.5.1 software. Fragmentation spectra obtained by LC-qTRAP MS/MS were examined in order to identify microginins present in the samples and to elucidate their structures.

## Declaration of Competing Interest

The authors declare that they have no known competing financial interests or personal relationships which have, or could be perceived to have, influenced the work reported in this article.
